# 3D Printing and processing of miniaturized transducers with near-pristine piezoelectric ceramics for localized cavitation

**DOI:** 10.1038/s41467-023-37335-w

**Published:** 2023-04-27

**Authors:** Haotian Lu, Huachen Cui, Gengxi Lu, Laiming Jiang, Ryan Hensleigh, Yushun Zeng, Adnan Rayes, Mohanchandra K. Panduranga, Megha Acharya, Zhen Wang, Andrei Irimia, Felix Wu, Gregory P. Carman, José M. Morales, Seth Putterman, Lane W. Martin, Qifa Zhou, Xiaoyu (Rayne) Zheng

**Affiliations:** 1 Department of Materials Science and Engineering, University of California, Berkeley, Berkeley, CA 94720 USA; 2 Department of Civil and Environmental Engineering, University of California, Los Angeles, CA 90095 USA; 3 Department of Mechanical and Aerospace Engineering, University of California, Los Angeles, CA 90095 USA; 4 Alfred E. Mann Department of Biomedical Engineering, University of Southern California, Los Angeles, CA 90089 USA; 5 Department of Ophthalmology, University of Southern California, Los Angeles, CA 90089 USA; 6 Leonard Davis School of Gerontology, University of Southern California, Los Angeles, CA 90089 USA; 7 Materials Technology R&D, Vehicle Technologies Office, Energy Efficiency and Renewable Energy, U.S. Department of Energy, Washington, DC 20585 USA; 8 Ronald Reagan UCLA Medical Center, University of California, Los Angeles, CA 90095 USA; 9 Department of Physics and Astronomy, University of California, Los Angeles, CA 90095 USA; 10 Present Address: Systems Hub, The Hong Kong University of Science and Technology (Guangzhou), Guangdong, 511453 China; 11 Present Address: College of Materials Science and Engineering, Sichuan University, Chengdu, 610064 China

**Keywords:** Sensors and biosensors, Mechanical engineering, Techniques and instrumentation, Ceramics

## Abstract

The performance of ultrasonic transducers is largely determined by the piezoelectric properties and geometries of their active elements. Due to the brittle nature of piezoceramics, existing processing tools for piezoelectric elements only achieve simple geometries, including flat disks, cylinders, cubes and rings. While advances in additive manufacturing give rise to free-form fabrication of piezoceramics, the resultant transducers suffer from high porosity, weak piezoelectric responses, and limited geometrical flexibility. We introduce optimized piezoceramic printing and processing strategies to produce highly responsive piezoelectric microtransducers that operate at ultrasonic frequencies. The 3D printed dense piezoelectric elements achieve high piezoelectric coefficients and complex architectures. The resulting piezoelectric charge constant, *d*_*33*_, and coupling factor, *k*_*t*_, of the 3D printed piezoceramic reach 583 pC/N and 0.57, approaching the properties of pristine ceramics. The integrated printing of transducer packaging materials and 3D printed piezoceramics with microarchitectures create opportunities for miniaturized piezoelectric ultrasound transducers capable of acoustic focusing and localized cavitation within millimeter-sized channels, leading to miniaturized ultrasonic devices that enable a wide range of biomedical applications.

## Abstract (web summary)

Despite advances in additive manufacturing of piezoceramics, resultant transducers generally suffer from high porosity, weak piezoelectric responses, and limited geometry. Here, authors report the design and printing of fully packaged freeform ultrasonic transducers capable of traveling inside mm-sized channels and deliver localized high ultrasound energy.

## Introduction

Owing to the capability of piezoelectric materials to convert mechanical to electrical energy and vice versa, they are widely used in sensing^1^, actuation^2^, energy harvesting^3,4^, cleaning^5^, and ultrasound imaging^6,7^. Recently, the emergence of new structural designs and computations has led to the prediction that incorporating 3D microfeatures into piezoelectric materials could provide unprecedented properties or functionalities, including designed anisotropy^8^ and the ability to emit tailored and localized ultrasound fields^9^, as well as sensors and actuators for miniaturized robots and transducers. The manufacturing of these architectures is either dependent on conventional machining methods, including etching, dicing and hot pressing^10^ due to the brittle nature of piezoelectric ceramic^11–14^, or limited to 3D printed composite materials containing piezoelectric nanoparticles and polymer matrices^15^. The mechanical stress caused by the machining processes results in grain pullout, reduced strength and depolarization, leading to significant degradation of the piezoelectricity of the manufactured elements^12^. As a result, the piezoelectric materials with these architectures exhibit piezoelectric coefficients much lower than those of their pristine ceramic counterparts and weak emission pressures when used as ultrasonic transducers. Printing piezoceramic transducer elements with precise microscale features, free form factors and high piezoelectric responses capable of acoustic focusing, impedance matching and serving as a backing layer is highly desirable in applications that require small sizes, high emission pressures and localized energy outputs that could open a myriad of new applications, including in situ imaging^16^, sonogenetic cellular modulation^17–19^, intravascular thrombolysis^20^, blood-brain barrier disruption^21^, neuromodulation^22,23^, and enhanced drug delivery via cavitation^24^.

An avenue addressing the challenge relies on precision additive manufacturing (AM) processes such as light-based stereolithography (SLA) approaches^25^ or two-photon lithography^26^ and post-processing (sintering) of the printed part^27^. During the SLA process, piezoelectric nanoparticles are mixed with photosensitive monomers to form composite colloids, which are used for UV curing to construct 3D composite elements in a layer-by-layer manner. The viscosity of the highly particle-loaded composite colloids makes printing a uniform layer difficult, and the light scattering effect^28^ of the particles induces broadening of the printed features, making printing precise features challenging. To generate dense piezoceramics, the as-fabricated composites (green parts) are sintered at high temperature to burn off the polymer matrices and regrow the ceramic grains. While piezoceramics, including sodium potassium niobate (KNN)^29^ and barium titanate (BTO)^30^, are most commonly used for 3D printing, the printing of lead-based piezoceramics, featuring a high piezoelectric constant and affordability, remains elusive because of the lead evaporation that occurs during the high-temperature sintering process^31^, which suppresses the functional performance of the sintered elements. Moreover, the conventional sintering process induces deformation, cracks, and high porosity in the 3D printed elements, resulting in degraded mechanical and piezoelectric properties.

Here, we present an SLA-based AM approach with an optimized post-sintering process for printing dense lead zirconate titanate (PZT) elements and packaged transducers with microscale features and a high piezoelectric response. Our process starts with high-resolution projection micro-stereolithography (PμSL) combined with a tape-casting recoating process to ensure accurate control of the features of the green parts. Inspired by the liquid phase sintering (LPS)^5^, which implements liquid phase of sintering additives during sintering to promote grain growth and improve the sintering behavior of ceramics, we incorporated a liquid phase sintering method compatible with SLA-based 3D printed PZT samples and implemented a liquid sealing method^32^ to suppress lead evaporation at high temperature, which reduces the porosity and lead loss of sintered elements. The as-fabricated PZT elements reached a piezoelectric charge constant and an electromechanical coupling factor of up to 583 pC/N, corresponding to 92.5% of that of the pristine material, outperforming currently printable piezoelectric materials.

In addition to active materials, we developed printable packaging material pallets that include a backing layer, an impedance matching layer and physical housing with a large range of tailorable impedances and attenuation coefficients to ensure optimal target performance for targeted applications. To demonstrate the applications of our approach, we printed a miniaturized ultrasound transducer with microscale focusing features, which is capable of generating high and localized acoustic pressure in blood vessels with diameters as low as 2.5 mm, allowing localized cavitation triggering, enhanced drug delivery, and ultrasonic modulation of cellular activity. The 3D printable microscale features together with the high piezoelectric properties of the printed piezoceramic materials will make a great leap forward toward new applications of 3D printed transducers.

## Results and discussion

### 3D printing of a highly loaded PZT composite and liquid phase sintering

**Fig. 1:**
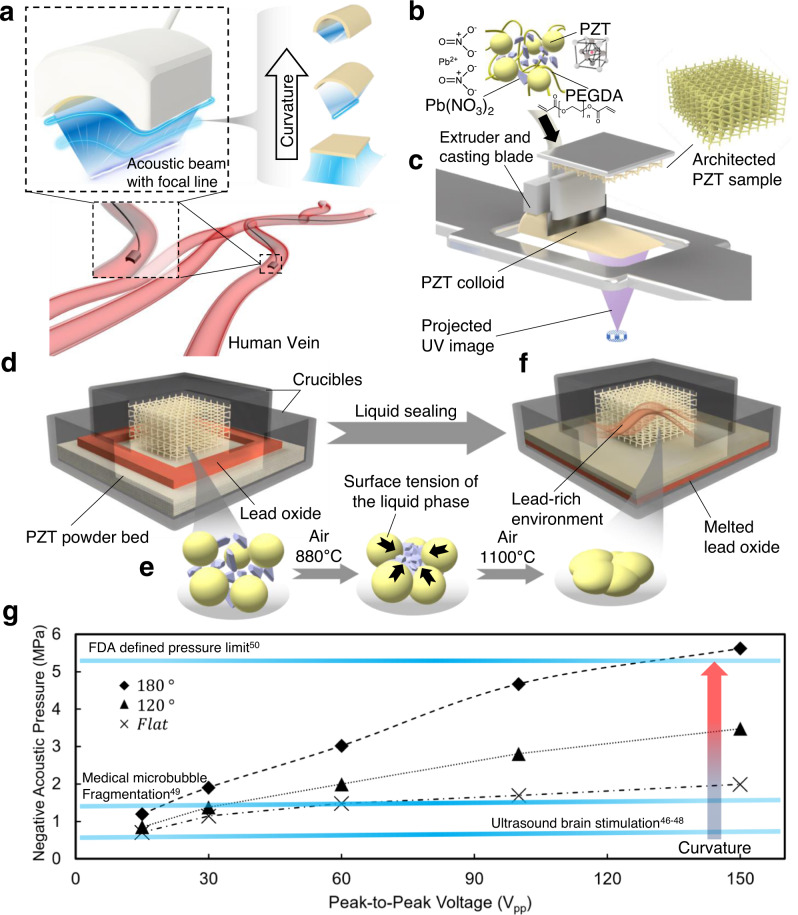
Miniatured ultrasound transducer design and 3D printing of a highly loaded PZT composite and liquid phase sintering. **a** Schematic of the fabricated miniatured ultrasound transducer with curved PZT elements. **b** Schematic of the liquid sintering resin components. **c** Novel 3D printing system for liquid phase sintering piezoelectric composites. **d** Debonding process to burn off the supportive polymer. **e** LPS process to form dense PZT sample. **f** Liquid sealing process to reduce the lead loss during high-temperature sintering. **g** Generated acoustic pressure curves of the 3D printed 9.75 MHz miniaturized ultrasound transducers, indicating that the values exceeded many medical thresholds at 9.75 MHz.

The free-form fabrication achievable with 3D printing technology allows us to exploit acoustic pressure and focusing capabilities with ranges and resolutions that are not feasible with conventional piezoelectric transducers consisting of flat piezoelectric elements (SI 6, Fig. S7). The miniatured ultrasound transducer is widely used for blood vessel disease diagnosis and treatment. However, currently existing miniatured ultrasound transducers have limited focusing or acoustic energy emitting capability^33–36^ due to the inability to fabricate high-resolution focusing features. Herein, we manipulate the generated acoustic beam of the ultrasound transducers by tuning the PZT element curvature. This curved shape leverages the ability to focus emitted acoustic energy along one line to improve the element sensitivity and acoustic beam resolution, as shown in Fig. 1a.

Our fabrication starts with UV-sensitive piezoelectric preparation. The highly particle-loaded slurry consists of 15~45 vol% PZT nanoparticles (APC 855), 5~15 vol% liquid phase sintering (LPS) agent, lead nitrate (Pb(NO_3_)_2_) and a UV-sensitive resin made from a mixture of monomers (polyethylene glycol diacrylate, PEGDA) and a photoinitiator (Methods), as shown in Fig. 1b. Compared to other PZT nanoparticles, APC 855 features a high piezoelectric charge constant (630 pC/N), which ensures the high performance of the printed part. LPS has been widely used in piezo ceramic fabrication^37,38^ and has been implemented in powder-based 3D printing techniques of piezoelectric materials^39^. However, incorporating LPS for light-based additive manufacturing is challenging as most sintering additives (lead oxide^38^ and CdBiB^40^) introduce additional light absorbance and increases viscosity, thus decrease the slurry printability. In this study, we used lead nitrate as the LPS agent due to its low UV light absorption, ensuring the high printability of the slurry. High PZT loading in the slurry could improve the piezoelectric properties but limited the printability. Here, we chose the combination of 35 vol% PZT and 5 vol% lead nitrates, which was experimentally optimized to guarantee the printability and piezoelectrical properties.

As illustrated in Fig. 1b, c, the slurry was fed to the resin tray of a custom-made PμSL system for 3D printing (Methods). A printing window (2 mm) made of polydimethylsiloxane (PDMS) was utilized during the process of separating the printed part and the printing window, which prevents small features from being damaged. The oxygen permeability of the PDMS window leads to an oxygen inhibition layer between the printing window and resin that increases the distance between the printed part and printing window and reduces the suction force^41^. In addition, compared to other printing windows, including fluorinated ethylene propylene (FEP) film^42^, acrylic board^43^ and glass window^44^, the high compliance of the PDMS window results in a slower separation process and protects the high precision features of the printed parts.

The as-printed piezocomposites were then sintered to grow the PZT grain size and form the dense PZT ceramic with designed geometries. First, the printed sample was debonded using a two-step debonding process (Methods, Fig. S1) to remove the supportive polymer, as shown in Fig. 1d. The two-step debonding process reduced the deformation of the printed elements compared to the currently existing direct debonding process, allowing fabrication of small-scale features. During the debonding process, lead nitrate within the elements decomposes into lead oxide at around 470 °C. After debonding, extra PZT powders were placed on the bottom of an alumina crucible, and the printed element was placed on top of the powder bed. A smaller crucible was used to cover the element and pushed down into the powder bed. The temperature was then increased to 1100 °C in 1.5 h and held for 3 h to grow the PZT grains and form a dense ceramic (Methods, Fig. S2a and S2b). LPS started at an elevated temperature. Lead oxide melted at 888 °C and contracted the PZT particles via surface tension, densifying the PZT architecture, as shown in Fig. 1e. The lead oxide inside the element also serves as a lead source during sintering to prevent lead loss^45^. The liquid sealing method was implemented by placing lead oxide along the edge of the small crucible (Fig. 1f) to further reduce the lead loss at high temperatures. During the sintering process, lead oxide melted, sealed the crucible, and created a lead-rich environment to avoid lead loss during the high-temperature sintering. Then, the samples were cooled down to room temperature at a rate of 10 °C/min. The sintered PZT material kept perovskite structure after high-temperature heat-treatment (Fig. S6c).

The sintering was followed by a polarization process to align the dipoles within the sintered PZT element and activate the piezoelectric effect (Methods, Fig. S3). The polarization was conducted in an isolation liquid (silicone oil) to prevent the breakdown in air with a polarization electric field higher than 3 V/μm. Based on the Curie temperature and breakdown electric field (6.5 V/μm) of the sintered PZT elements, we optimized the temperature and electric field profile of the polarization process (Fig. S3c). After reaching the peak value, the electric field was maintained during the cooling process of the isolation liquid to prevent depolarization of the printed element.

Our optimized sintering procedure ensured the formation of a dense ceramic with low porosity and well-maintained lead content, leading to high piezoelectric charge constant and coupling factor. The acoustic pressure generated by the fabricated 9.75 MHz transducer can be effectively increased by using the curvature design of the elements (Fig. 1g). Notably, the acoustic pressure is tunable according to the change in the element curvature degree and can exceed some medical thresholds^46–50^ at 9.75 MHz (Method), enlarging the range of applications of the fabricated device.

### Piezoelectric performance of the as-fabricated piezoelectric elements

**Fig. 2:**
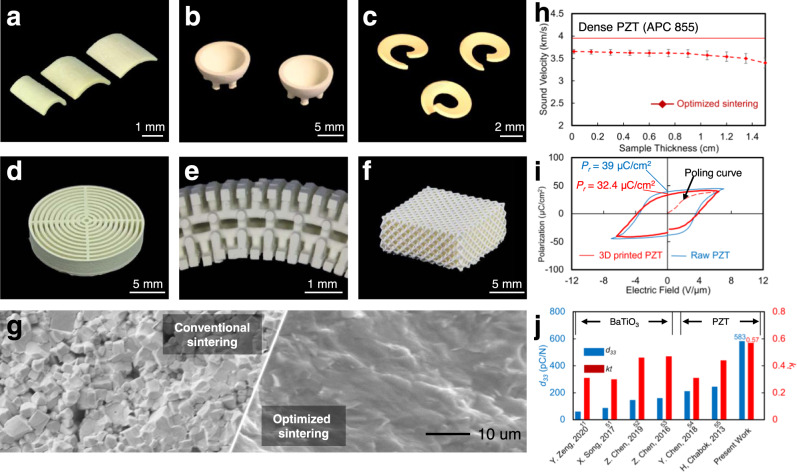
PZT free-form fabrication and piezoelectric performance of the as-fabricated piezoelectric elements. **a** Micro-curved stave elements with different curvatures. **b** Hemisphere elements used in a general ultrasound transducer for medical imaging and nondestructive testing. **c** Helical element for generating spiral acoustic fields for ultrasound manipulation. **d** Cylindrical transducer element with multiple concentric annular layers. **e** Lattice sensor element for low-frequency (<100 kHz) ultrasound. **f** Force sensor element with ultrahigh sensitivity. **g** SEM image comparison between samples sintered by conventional sintering and optimized sintering methods. **h** Sound speed measurements against the sample thickness. Error bars represent standard deviation (*n* = 5). **i** Polarization-electric field (P-E) loop of 3D printed PZT. The remnant polarization *P*_*r*_ is 32.4 µC/cm^2^, which is 84% of the value of pristine materials. **j** *d*_*33*_ and coupling factor *k*_*t*_ benchmar*k*ed with state-of-the-art 3D printed piezoelectric materials.

Our technique allows the fabrication of high-performance piezoelectric transducers with complex 3D geometries that are not achievable by any conventional fabrication methods for piezoceramics, including hot-pressing, molding, sanding, and dicing. These complex shapes allow for potential custom transducer applications. Figure 2a shows curved elements with designed curvatures for potential miniatured transducer applications. Other examples include hemisphere elements (Fig. 2b) for medical imaging and nondestructive testing, helical elements (Fig. 2c) for generating acoustic beams with vortex motion for ultrasound manipulation^9^, cylindrical transducer elements with multiple concentric annular layers filled with a polymer (Fig. 2d) for reducing the traverse vibration and enhancing the thickness vibration, and architected piezo sensors (Fig. 2e, f) for underwater sensing.

To quantify the deformation induced during the sintering process, we measured the linear shrinkage and curvature change of the element after sintering. The results showed an average linear shrinkage as high as 27.73% and a curvature change within 0.5% (SI 2 and Fig. S4). Figure 2g shows the SEM image comparison of an element between the conventional sintering method and the optimized sintering method, confirming the low porosity of the as-fabricated elements. The density of the sintered elements was measured on a set of PZT disks (diameter 5 mm, thickness 390 μm, *n* = 5). The density of the sintered elements reached 7.2 g/cm^3^, which is 94.7% of the pristine material (APC 855). The porosity of the elements, 5.3%, is calculated from the density of the printed samples. The sound speed within both the 3D printed element and its pristine material counterpart were measured within multiple PZT plates with the same cross-section area (5 × 5 mm) and different thicknesses to verify the low porosity of samples with different volumes. The sound traveling time in each element was captured to calculate the sound speed (SI 3, Fig. S5). As shown in Fig. 2h, the sound speed in the 3D printed samples reaches approximately 93.2% of the speed in the pristine material, which is consistent with the density measurements.

The piezoelectric properties (hysteresis loops, piezoelectric charge constant, *k*_*t*_) of the as-fabricated PZT elements were measured to compare with the pristine materials. Figure 2h plots the hysteresis of the polarization of the PZT element measured by precision multiferroic analyzer (Radiant Technology. Inc, USA), showing a remnant polarization (*P*_*r*_) as high as 32.4 µC/cm^2^ (Methods). The strain-electric field loops of the 3D printed and the pristine elements were captured (Methods, SI 4, Fig. S6), showing that the 3D printed sample can generate near-pristine strain under the same electric field. The piezoelectric charge constant, *d*_*33*_, was measured using a *d*_*33*_ meter (YE2730A, APC Piezo, USA) and reached 583 pC/N, 92.5% of the value of the pristine material. The electromechanical coupling factor in the thickness direction, *k*_*t*_, was measured as 0.57 (Methods, Fig. S9). Figure 2j compares the *d*_*33*_ and *k*_*t*_ of our 3D printed PZT with those of the state-of-the-art 3D printed piezoceramics^11,51–55^.

### Packaging

**Fig. 3:**
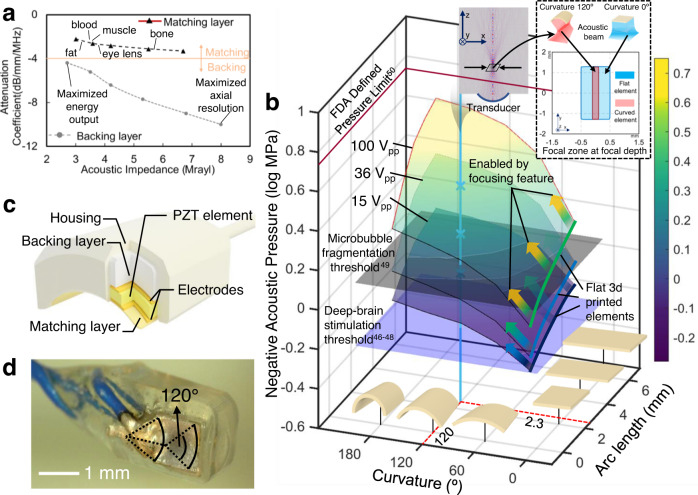
Packaging design and acoustic energy output performance of the as-fabricated miniaturized ultrasound transducer. **a** 3D printable materials with tunable attenuation coefficient and acoustic impedance for transducer packaging. **b** Acoustic pressure map vs. 3D printed miniaturized transducer curvature and arc length, showing that the generated acoustic pressure already exceeds many medical application thresholds. **c** Schematic image of a 3D printed miniaturized ultrasound transducer. **d** Optical image of a 3D printed miniaturized ultrasound transducer with a focused micro-PZT element.

The acoustic signal resolution and energy output of ultrasonic transducers highly depend on the packaging due to the mismatch of acoustic properties of the ceramic transducer element and transmission medium. To achieve optimal resolution and acoustic energy output, the acoustic properties of the matching layer and backing layer of the transducer package need to be optimized for target applications. In addition, the microscale features and complex shapes of a 3D printed transducer require conformal packaging to ensure tight contact with and encapsulation with the transducer element. Herein, we developed 3D printable composite material pallets (Methods) with acoustic impedance ranging from 3 Mrayl to 6.7 Mrayl and attenuation coefficient ranging from −4.3 dB/mm to −10 dB/mm, as shown in Fig. 3a. The acoustic properties of the materials can be tuned by changing the ratio of the resin composites. The matching layer composite (SI 5), featuring low attenuation coefficient and a large range of acoustic impedances, was used as the matching layer, while a backing layer composite (SI 5), featuring a large range of attenuation coefficients, was used as the backing layer. The large tunable range of impedances and attenuation coefficients allows us to fabricate transducers with optimal performances for targeted applications. To maximize the bandwidth, acoustic wave transmissivity and output pressure, the acoustic impedance (*Z*_*m*_) of a single matching layer should follow the rule^56^:

1 $${Z}_{m}={\left(Z{Z}_{1}^{2}\right)}^{1/3}$$

Where *Z* and ${Z}_{1}$ are the piezo element acoustic impedance and medium acoustic impedance, respectively. Figure 3a demonstrates the optimal matching layer composition when the transducer is used in different tissues, including blood, muscle, fat, and bone. The selection of the backing layer depends on the applications. For acoustic imaging, as an example, the backing layer requires high attenuation to prevent the ringing effect^57^. For high acoustic pressure output, a large impedance mismatch between the backing layer and the transducer is required to maximize the bounce-back pressure^6^. Moreover, during in vivo operation, the encapsulation material of the transducer is directly in contact with the tissue and requires biocompatibility. We used a biocompatible material, trimethylolpropane triacrylate (TMPTA), as the encapsulation material to minimize the damage to the tissues. The energy output performance variation of the representative packaged transducers along with their geometries are discussed in SI 7 and Fig. S8.

### 3D printed focused ultrasonic transducers

We show the effect of transducer shape factors, including curvature and arc length, and the driving voltage on the acoustic pressure of the transducer. As shown in Fig. 3b, piezo element curvatures ranging from 0° to 180° and arc lengths ranging from 1 mm to 6 mm, which fit a typical portal vein inner diameter (6–12 mm)^58^, were simulated in COMSOL Multiphysics for different voltage inputs. The width of the simulated elements was 2.5 mm to ensure steerability of the transducer inside a blood vessel. The bold curves with a curvature equal to zero denote the flat piezoelectric transducers with limited acoustic pressure outputs, while the surface plots denote the output pressure range enabled by the 3D printed curved elements. The focusing feature enabled the transducer to minimize its focal zone by 78.6%, as shown in the subfigure in Fig. 3b. The results show that the miniaturized ultrasound transducer with 3D printed focusing features reaches higher pressure and lateral resolution than the reported ultrasound transducers (SI 6, Fig. S7). Remarkably, the 3D printed focusing elements reach the FDA defined pressure limit^50^ and many other medical threshold^46–49^ at 9.75 MHz. The highly localized acoustic pressure may open the door for applications for targeted ultrasonic therapy, such as non-contact cellular modulation, localized drug delivery, and transient blood-brain barrier opening in small areas with length scales under 2 mm.

The acoustic pressures were then experimentally verified with an element with a curvature of 120° and an arch length of 2.3 mm. The schematic (Fig. 3c) of the miniaturized ultrasound transducer shows the package component assembly used for experimental verification. To maximize the pressure output in blood, using the chart in Fig. 3a, we chose the optimal matching layer (CF (28 wt%) - matching layer composite) and backing layer (Fe (3 wt%) - backing layer composite). Figure 3d shows an optical image of the 3D printed miniaturized ultrasound transducer. Two copper cables (300 mm length, 0.5 mm diameter) were used to connect the device electrodes with the power supply and allow the transducer to be placed in veins. The as-fabricated piezo element was designed with a resonant frequency of 9.75 MHz, which is ideally suited for high resolution focusing and high output pressure in the usual range of the medical ultrasound for procedure guidance^59,60^. 9.75 MHz burst waves were used to drive the miniaturized ultrasound transducer (Methods). The output pressures of the transducer with different voltage inputs were captured by a commercial hydrophone (HGL-0400, OND) in DI water and are plotted in Fig. 3b (with X shaped dots), which well match the simulated results.

### Underwater cavitation generated by the as-fabricated transducer

The highly localized acoustic output of the 3D printed transducer allows us to exploit its applications for localized cavitation. Cavitation refers to the process in which vapor- or gas-filled cavities formation and undergo oscillation or growth and implosion in liquids upon exposure to acoustic radiation^61^. It can generally be classified into two types: stable cavitation and inertial cavitation. Stable cavitation^62^ is identified by sustained small amplitude oscillations of the cavities at about their equilibrium, which induces microstreaming in the liquid. Inertial cavitation is characterized by the process of cavities growth and collapse, which induces microjets, causing pitting on solid surface^61^. In biomedical applications, inertial cavitation disrupts the structure of the carrier vesicle and triggers the release of drugs; the induced microstreaming and microjet also makes cell membranes and capillaries more permeable for drug transportation from the blood, making the cavitation a powerful tool for localized endogenous drug delivery. However, triggering cavitation usually requires high acoustic pressure. The cavitation and the triggering ultrasound can cause collateral damage, including tissue burning, hemolysis and blood temperature increase^61–64^. Additionally, the treatment of diseases such as cancer, degenerative and inflammatory diseases, or thromboembolic diseases requires a high concentration of certain drugs to be delivered that have toxic side effects on other tissues^24^. Precise control and localize cavitation at target tissue sites is desired to minimize those side effects. Using microbubble as the cavities can effectively induce the cavitation, making it more controllable^20^. But the localized cavitation induced by the microbubble oscillation or fragmentation requires precise delivery of acoustic pressure in a controlled manner within a confined volume as small as hundreds of microns, which cannot be achieved by conventional miniatured ultrasonic elements without focusing features.

Inspired by this challenge, we demonstrate our 3D printed high-pressure miniaturized ultrasound transducer with focusing features to precisely burst microbubbles and generate cavitation. The piezo element has a curvature of 120° and an arch length of 2.3 mm, yielding a focal depth of 0.9 mm. The center frequency of the fabricated transducer is 9.75 MHz, as shown in the pulse-echo signal frequency spectrum (Methods, Fig. S10).

**Fig. 4:**
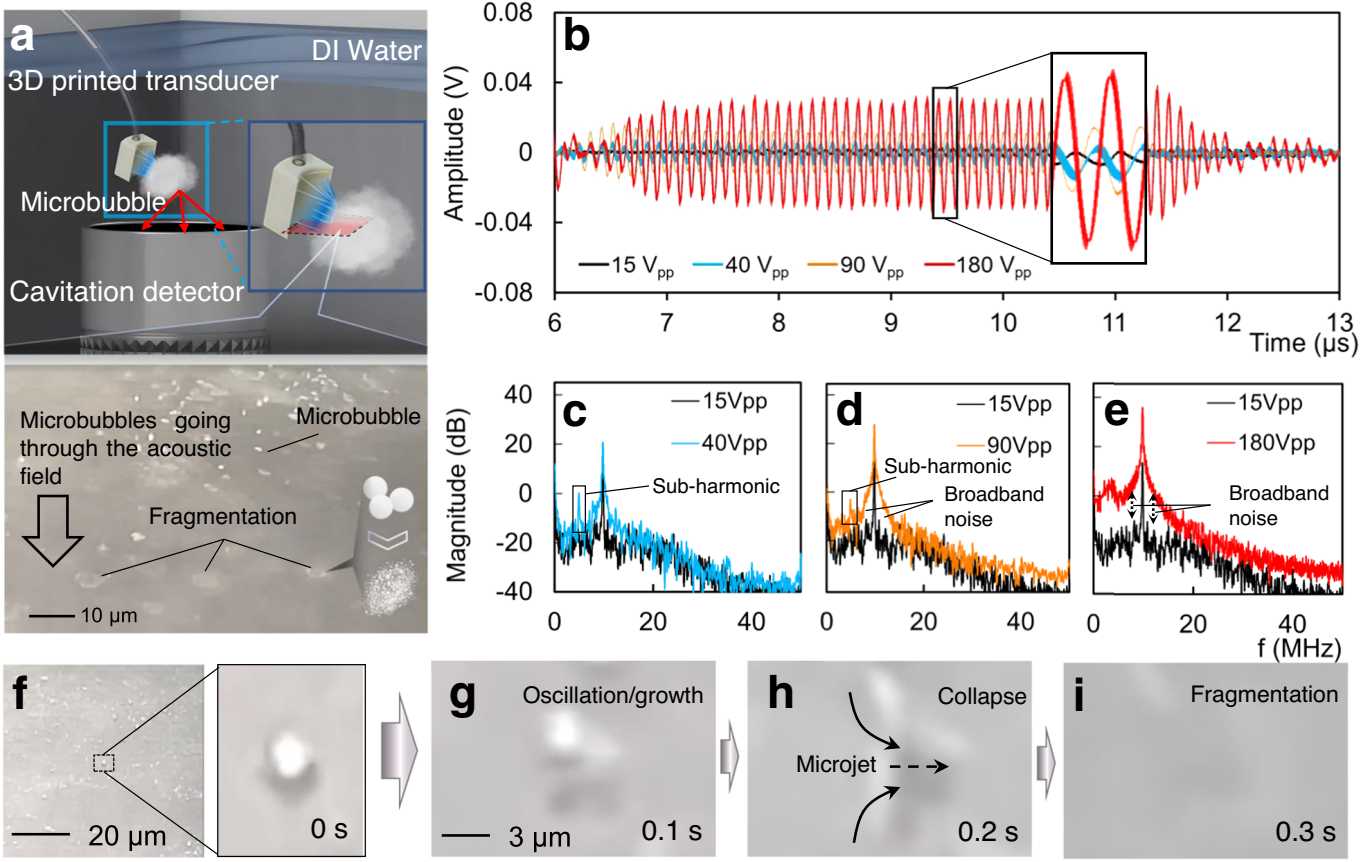
Microbubble-induced cavitation. **a** Schematic of the experiment on capturing the cavitation signal in a microbubble suspension and zoom-in view of microbubble fragmentation. **b** Captured signals (Input voltage: 15 V_pp_, 40 V_pp_, 90 V_pp_ and 180 V_pp_) in time domain. **c–e** Frequency spectrums of the received signals driven by different input voltages. The sub-harmonic and broadband are identified as the generation of stable cavitation and inertial cavitation, respectively. **f–i** Microbubble behavior during insonation, showing the processes of oscillation, growth, and collapse of a microbubble.

To verify the cavitation generated by the 3D printed transducer, 25 mg microbubbles (Lumason, BRACCO, USA) were diluted in a 5 ml 0.9% sodium chloride injection and burst by the 3D printed transducer driving by 9.75 MHz burst waves with 50 cycles (Method). We adjusted the input voltage of the transducer from 15 V_pp_ to 180 V_pp_ and captured the ultrasound signal with a cavitation detector (Sonic Concepts Inc., Bothell, WA, USA) in a large water tank (free field), as shown in Figs. 4a, b. The captured signals were transformed (Fast Fourier Transform) into frequency spectrums to verify cavitation (Fig. 4c–e). As shown in Fig. 4c, with a 40 V_pp_ excitation, the spectrum clearly shows the sub-harmonic of the fundamental frequency (9.75 MHz), which is an indication of the stable cavitation generation^65^. As the input voltage increases, both broadband noise and sub-harmonic were captured (Fig. 4d), indicating that stable cavitation and inertial cavitation^66^ were activated by our transducer simultaneously. With a higher voltage, the sub-harmonic disappeared while and the broadband noise increased by 40 dB (Fig. 4e) compared to that of 15 V_pp_ excitation, showing the microbubbles were undergoing stronger inertial cavitation^67^.

The process of microbubble fragmentation was captured by an inverted microscope (IX71, Olympus America Inc., Center Valley, PA, USA). As shown in Fig. 4f and Supplementary Movie 1, the microbubbles fragmented and collapsed in the focal area when the transducer was excited by 9.75 MHz 180 V_pp_ pulses. The microbubble behavior during insonation was captured when transducer was driving by 50 V_pp_ pulses with 50 cycles (Fig. 4g–i, Supplementary Movie 1). The microbubbles experienced oscillation, growth, and collapse, indicating both stable cavitation and inertial cavitation can be generated by our 3D printed transducer^62^. The microbubble-induced cavitation shows the capability of the miniatured transducer to assist the drug delivery and diffusion (Cavitation-assisted dye diffusion is shown in SI 8, Fig. S11, Fig. S12 and Supplementary Movie 2).

### Localized cavitation

**Fig. 5:**
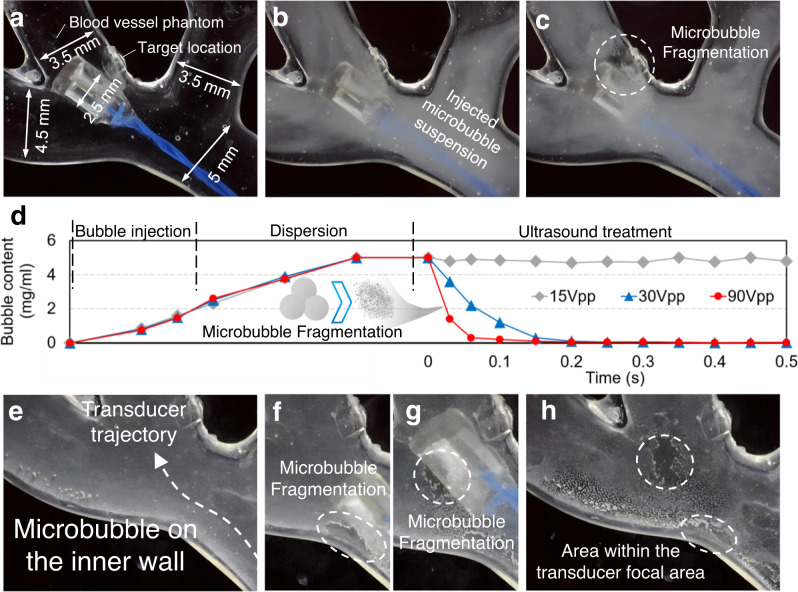
Miniaturized ultrasound transducer-induced localized cavitation in blood vessel phantom. **a–c** Ultrasound-induced microbubble fragmentation via our 3D printed miniaturized ultrasound transducer in a 3D printed blood vessel phantom. **d** Microbubble content vs. time, indicating that the fragmentation rate is related to the input voltage. **e–h** Localized drug delivery testing. The microbubbles can be controlled to fragment only at the focal area.

To demonstrate the potential of our transducer for precision transvascular drug delivery via low sound pressure cavitation, we prepared a 3D printed transparent blood vessel phantom with multiple internal channels to mimic the in vivo physical environment and inject the transducer and microbubbles inside it. As shown in Fig. 5a, the transducer was focused on the targeted location. During this time the microbubble suspension (5 mg/ml) was injected into the blood vessel phantom (Fig. 5b). Next, pulses (Methods) were applied to the transducer to generate acoustic pressure. A transparent region appearing in the focal area (Fig. 5b, Supplementary movie 3) was caused by microbubble fragmentation and acoustic radiation force. Figure 5d plots the bubble content as a function of time captured by digital image correlation (Methods, SI 6, Fig. S13), showing that the bubbles were fragmented within 0.15 s and 0.08 s for 50 Vpp and 90 Vpp driving voltages. With a driving voltage lower than the cavitation threshold, no microbubble fragmentation observed. To demonstrate the high precision focusing, we deposited bubbles on the internal wall of the vessel phantom (Fig. 5e) and used the transducer to fragment the bubbles. As shown in Fig. 5e–h and Supplementary movie 4, as the transducer moved, only the bubbles within the focal area (0.2 mm^2^) were fragmented, showing high-precision localized cavitation.

In summary, via a combination of high-resolution piezoceramic matrix printing and a liquid agent-assisted sintering approach, we demonstrate a 3D printed microstructured transducer with near-pristine piezoelectricity, which enabled the delivery of ultrasound to focal areas confined within areas on the order of sub-mm^2^ and achieved target cavitation and cavitation-enabled drug delivery. Liquid phase sintering and sealing reduced the PZT lead loss, sample porosity, and the deformation. The piezoelectric properties of the materials such as the *d*_*33*_ and *k*_*t*_ outperformed those of existing 3D printed piezoceramics. We studied the effect of micro-structures and curvatures enabled by the printing approach on the focusing resolution and power output and optimized the spatial resolution and acoustic pressure of the output acoustic beam. Additionally, the 3D printed packaging components with tunable acoustic properties adapt the PZT elements to different transmission media and applications, diversifying the versatility of the fabricated ultrasound transducers in medical treatments.

The reported processing approach achieves the high energy output application of an ultrasound transducer driven by a 3D printed piezo element. Optimization of the microstructural effect, along with tunable matching and backing layer properties, enables high acoustic pressure confined within sub-mm^2^. Beyond the drug delivery, the localized acoustic energy output could enable future applications including intravascular thrombolysis, in situ imaging, neuromodulation, sonogentic control, and oncology applications.

## Methods

### Fabrication of the 3D piezoelectric nanocomposites

The fabrication process started with the preparation of the UV-sensitive piezocomposite colloid consisting of PZT 855 (APC Piezo, USA) particles, lead nitrate (MilliporeSigma, USA) and the UV-sensitive resin. The UV-sensitive resin was comprised of 1 wt% to 3 wt% photoinitiator Irg819 (MilliporeSigma, USA) and PEGDA (MilliporeSigma, USA). 15 vol% to 45 vol% of PZT, 5 vol% to 15 vol% lead nitrate was ball milled together with the UV-sensitive resin to form the colloid. These materials were subsequently used for printing on a high-resolution projection micro-stereolithography system integrated with the tape casting method^8^. The designed 3D structure was created in Autodesk Inventor (Autodesk, inc. USA), and then sliced into 2D images with Netfabb (Autodesk, inc. USA) for the printing process. During the printing, the colloid was coated on the printing window with one layer thickness by controlling the height of the casting blade^8^. One slice of the printing model was projected by the UV projector (light power intensity: 14.8 mW/cm^2^) onto the printing window and used to solidify one layer of the colloid. The solidified layer was attached to the building platform, or the previous layer and the printing stage was lifted from the printing window, preparing for the coating and curing process of the next layer. The piezoelectric composite with designed 3D geometry was fabricated by repetition of the coating and UV curing processes. The printing resolutions in x-y plane and along z-axis were 20 μm and 15 μm, respectively (details in SI 1).

### Debonding and sintering

The two-step debonding process is shown in Fig. S1a. The debonding temperature profile is shown in Fig. S1b. Firstly, the printed sample was heated under an argon environment. The temperature was increased from 0 °C to 600 °C at a rate of 1 °C/min in 10 h, and held at 600 °C for three hours, during which the supportive polymer (PEGDA) was carbonized and utilized to maintain the shape of the PZT composite structure. Then, the carbonized polymer was burnt out in the air at 600 °C for 3 h. The two-step debonding process reduced the deformation of the printed elements compared to the direct debonding process^54^, allowing the fabrication of small-scale features.

After debonding, the temperature increased to 1100 °C for grain growth and dense ceramic formation. The peak sintering temperature was optimized according to the *d*_*33*_ testing results and sample breakdown electric field. With a peak sintering temperature higher than 1100 °C, the *d*_*33*_ dropped due to lead loss caused by high temperature (Fig. S2a). When the peak sintering temperature was lower than 1100 °C, the sample would have a low density, resulting in a low breakdown electric field (Fig. S2b).

### Polarization

The polarization process was conducted in silicone oil to avoid electric breakdown when the polarization field is over 3 V/µm, which is the breakdown field of the air. Both surfaces of the sample were covered by silver electrode plates wired to the high voltage supply, as shown in Fig. S3a. The piezoelectric sample was polarized under 6.5 V/μm electric field, resulting in dipole alignment in the direction of the applied field (Fig. S3b). A combination of an electric field profile and a temperature profile was used to polarize the as-fabricated piezoelectric nanocomposite, as shown in Fig. S3c.

### Hysteresis loops measurement

The P-E loop was measured by Precision Multiferroic Analyzer from Radiant technology. Oil drops were applied to the sample to prevent electric breakdown. The S-E loop measurement was performed by Thin Film Piezoelectric Test Bundles (Radiant technologies, Inc, USA). The testing bundle consists of a precision materials analyzer (Precision Multiferroic II, Radiation technologies, Inc) and a laser vibrometer (VibroOne, Polytec, Inc. USA) (details in SI. 4, Fig. S6). The tested samples had the same dimensions (2.5 × 2 × 0.25 mm). In S-E loop testing, the samples were polarized under 6.5 V/μm electric fields using the same poling method described in the manuscript.

### Electromechanical coupling factor

The electromechanical coupling factor *k*_*t*_ was calculated using resonance frequency *f*_*r*_ and anti-resonance frequency *f*_*a*_^68^ via:

2 $${k}_{t}=\sqrt{\frac{\pi {f}_{r}}{2{f}_{a}}\times \cot \frac{\pi {f}_{r}}{2{f}_{a}}}$$

*f*_*r*_ and *f*_*a*_ were extracted from the impedance curve measured using an E4991B impedance analyzer (Keysight Technology, USA). The spectrum of impedance of the as-fabricated elements (2.1 × 1.9 × 0.15 mm) is shown in Fig. S9. The resonant frequency and anti-resonant frequency were 8.27 MHz and 9.77 MHz, respectively, leading to an electromechanical coupling factor of 57.2%.

### Equivalent medical threshold calculation

The equivalent thresholds at the working frequency of our transducer in this study was calculated using M.I. value^50^ via:

3 $$M.I.=\,\frac{{p}_{{negative}}}{\sqrt{f}}$$

where *p*_*negative*_ is the peak negative pressure and $f$ is the ultrasound wave frequency.

### Ultrasound setup

A function generator (SDG1025, SIGLENT, China) was connected to a radio frequency power amplifier (100A250A, Amplifier Research, USA) to drive the transducer. 9.75 MHz burst waves with 50 cycles (20 ms burst period) were input into the transducer. For localized cavitation experiments in blood vessel phantom, the 9.75 MHz burst waves were set up to have 10% duty cycle.

### Miniaturized ultrasound transducer pulse-echo signal

The pulse-echo profile of the as-fabricated transducer with optimized packaging is shown in the Fig. S10. JSR pulser receiver (DPR300, JSR Ultrasonics, USA) was used to drive the transducer and receive the echo signal. The frequency spectrum indicates that the transducer has the largest output at frequency is 9.75 MHz. The −6 dB bandwidth of the signal is 57%, which is also suitable for imaging application^69^.

### 3D printable packaging material

The 3D printable composite material pallets contain two resin composite systems for matching and backing layer of a transducer. The loaded fiber/particle were sonicated and uniformly mixed with the UV curable resin using ball mill. The acoustic properties (acoustic impedance, attenuation coefficient) of the cured composite can be tuned by adjusting the loading of fibers in the composites (details in SI 5). The 3D printable composite materials were printed using the same custom PμSL fabrication system as the PZT fabrication.

### Acoustic simulation

The acoustic simulations were conducted using COMSOL Multiphysics 6.0. The transmission medium was set to water. The boundaries of simulation models were set to perfect matching layer to avoid reflection. The materials’ properties (piezoelectric properties, acoustic impedance and sound speed) in the model were using the tested properties of our samples.

### Dye diffusion coefficient and microbubble concentration

The dye diffusion coefficient and concentration of microbubble in the container were calculated by a self-developed digital image correlation method based on the transparency of the solution. The screenshots of the target location were converted into gray scale image using MATLAB (MathWorks, USA). The mean pixel values of images were used for computing the dye diffusion speed and microbubble concentration change (details in SI 8, Fig. S11, S12 and S13).

### Reporting summary

Further information on research design is available in the Nature Portfolio Reporting Summary linked to this article.

## Supplementary information

Supplementary Information

Description of Additional Supplementary Files

Supplementary Movie 1

Supplementary Movie 2

Supplementary Movie 3

Supplementary Movie 4

Reporting Summary

## Data Availability

All data are available in the manuscript and in the supplementary information and the figshare database with the link 10.6084/m9.figshare.22186420^70^.

## Code availability

Numerical simulations in this work are performed using the commercial finite element software COMSOL Multiphysics and MATLAB. All related codes are available from the corresponding authors upon request.
