# Supplementary material for: 3D Printing and processing of miniaturized transducers with near-pristine piezoelectric ceramics for localized cavitation
Source: Nat Commun. 2023 Apr 27;14:2418. doi: 10.1038/s41467-023-37335-w (PMC10140030; doi:10.1038/s41467-023-37335-w)
Supplement: Supplementary file 1 — Supplementary Information [file 41467_2023_37335_MOESM1_ESM.pdf]

Supplementary Information for

3D Printing and Processing of Miniaturized Transducers with Near-pristine Piezoelectric Ceramics for Localized Cavitation

Author: Haotian Lu[1,2,3,†], Huachen Cui[2,10,†], Gengxi Lu[4,5], Laiming Jiang[4,5,11], Ryan Hensleigh[2], Yushun Zeng[4,5], Adnan Rayes[4,5], Mohanchandra K. Panduranga[3], Megha Acharya[1], Zhen Wang[1,2], Andrei Irimia[4,6], Felix Wu[7], Gregory P. Carman[3], José M. Morales[8], Seth Putterman[9], Lane W. Martin[1], Qifa Zhou[4,5], Xiaoyu (Rayne) Zheng[1,2,]*

Author address:

[1]Department of Materials Science and Engineering, University of California, Berkeley, Berkeley, California 94720, USA

[2]Department of Civil and Environmental Engineering, University of California, Los Angeles, California 90095, USA

[3]Department of Mechanical and Aerospace Engineering, University of California, Los Angeles, California 90095, USA

[4]Alfred E. Mann Department of Biomedical Engineering, University of Southern California, Los Angeles, California 90089, USA

[5]Department of Ophthalmology, University of Southern California, Los Angeles, California 90089, USA

[6]Leonard Davis School of Gerontology, University of Southern California, Los Angeles, California 90089, USA

[7]Materials Technology R&D, Vehicle Technologies Office, Energy Efficiency and Renewable Energy, U.S. Department of Energy, Washington, DC 20585, USA

[8]Ronald Reagan UCLA Medical Center, University of California, Los Angeles, California 90095, USA

[9]Department of Physics and Astronomy, University of California, Los Angeles, California 90095, USA

[10]Systems Hub, The Hong Kong University of Science and Technology (Guangzhou), Guangdong 511453, China

[11]College of Materials Science and Engineering, Sichuan University, Chengdu 610064, China

[†]These authors contributed equally to this work
[*]Corresponding author: Xiaoyu (Rayne) Zheng   Email: Rayne23@Berkeley.edu

This document include:

Supplementary Information 1 to 8
Supplementary Fig. S1 to S13
Table S1
References

SI. 1 Fabrication resolution of the 3D piezoelectric nanocomposites

During the curing process, the resolution of the custom PμSL fabrication system in x-y plane (20×20 μm) is determined by the pixel size of the projector. The resolution along z-axis is determined by the curing depth of colloid. The curing depth of the solid layer is given as[1]:

$$Z_{ct} = \frac{1}{\alpha} ln\left(\frac{E}{E_c}\right) \qquad S1.1$$

where, α is the resin absorption coefficient, E and E_c are the actual and critical exposure, respectively. In our fabrication process, the layer thickness was set as 15 μm, which is smaller than half of the cure depth to ensure tight bonding between two consecutive layer[2]. This optimized cure depth for each layer was modulated by tuning the exposure time[3]. A reductive lens was utilized to increase the fabrication resolution of the printed structure[3]. The range of achievable 3-3 vibration mode frequency of a 3D printed thin-film sample is determined by the range of thickness that can be achieved, which is around 10.8 μm. This corresponds to the highest achievable frequency on the order of 171 MHz. Higher frequency can be potentially achieved by reducing the minimal layer thickness of the printing apparatus (employing Z-stage with higher resolution).

SI. 2 Element shrinkage during sintering and its effects on the structural integrity

To evaluate the effects of shrinkage on structural integrity, we measured three types of geometrical parameters before and after sintering, including length change (characterized on flat samples), curvature deviation (measured on concave samples) and strut thickness deviations. Here, 20 rectangular samples (3×2.6×0.2 mm) were fabricated and the dimension change before and after sintering was measured, as shown in Fig. S4a-b. Fig. S4c shows the deviation distribution of the average linear shrinkage ratio with an average value of 27.73% and a standard variation of 1.9%, indicating a uniform shrinkage ratio among samples. Fig. S4d-e show the representative sample before and after sintering. The average curvature change is within 0.5° (0.51% of the original curvature) with a standard deviation of 2.2° (Fig. S4e-f). The resultant acoustic pressure change is within 0.04 MPa based on Fig. 3d, which is negligible in our experiments. Furthermore, we evaluated the strut thickness uniformity in 3D architectures. We used X-ray computed tomography (CT, Bruker microCT, Model Skyscan 1174) to capture the morphology of a sintered lattice sample containing 84 struts. Fig. S4i shows the deviation distribution of the strut thicknesses. The thickness of the sintered struts is less than 1.58% thicker than the as-designed value (300 μm) with a standard deviation of 6.6 μm, indicating that the structural integrity was well kept after sintering.

SI. 3 Acoustic properties of as-fabricated material

Measurement of acoustic impedance

The schematic of the setup that was used to measure sound speeds is shown in Fig. S5a. The sound speed in the material $V_{material}$ calculated using the phase change $\Delta\phi$ of the received signal after

inserting the material in between the two standard transducers (Fig. S5c) via:

$$V_{material} = \frac{tV_{water}}{t - \Delta\phi V_{water}} \qquad S3.1$$

where, t indicates the thickness of the material to be tested, V_{water} is the sound speed in water. The acoustic impedance is calculated by[4]:

$$Z = \rho V_{material} \qquad S3.2$$

where, ρ is the density of the material.

Measurement of attenuation coefficients

The transmission ratio, t_p, between the transmitted wave acoustic pressure and the incident wave acoustic pressure on the interface[5] can be represented by:

$$t_p = \frac{2Z_2}{Z_1 + Z_2} \qquad S3.3$$

where the Z_1 and Z_2 indicates the acoustic impedance of the two materials. Thus, the acoustic pressure p in the material near the interface I and interface II (Fig. S5b) are represented using the transmission ratio t_{p12} at interface I and t_{p34} at interface II via:

$$p_2 = t_{p12} p_1 \qquad S3.4$$

$$p_3 = t_{p34} p_4 \qquad S3.5$$

Since the acoustic pressure p has a linear relationship with the voltage V generated by the transducer for measuring, the acoustic pressure ratio between top and bottom side within material can be represented using the voltage amplitude V by:

$$\frac{p_3}{p_2} = \frac{(Z_1 + Z_2)^2 V_4}{4 Z_1 Z_2 V_1} \qquad S3.6$$

where, Z_1 and Z_2 are the acoustic impedance of the medium (water) and the material to be tested. V_1 and V_4 are the measured voltage generated by ultrasound transducer placed at the top and bottom side of the material. For a given frequency, converting the unit of S3.6 into (dB/mm) can yield the

attenuation coefficient of the material via:

$$\alpha = \frac{20}{d}\log\frac{(Z_1 + Z_2)^2 V_4}{4Z_1 Z_2 V_1} \qquad S3.7$$

where, d is the thickness of the material.

SI. 4 Strain – electric field curves of the as-fabricated material

For the S-E loop, we measured the strain in the thickness direction (3-3 mode) of the 3D printed material and a standard commercial material against the applied electric fields (S-E curves) using Thin Film Piezoelectric Test Bundles (Radiant technologies, Inc, USA) at room temperature (21.3 °C), as shown in Fig. S6a. The system consists of a precision materials analyzer (Precision Multiferroic II, Radiation Technologies, Inc) and a laser vibrometer (VibroOne, Polytec, Inc. USA). The precision materials analyzer applied voltage long the polarization direction (thickness direction) of the sample and the displacements of the samples were collected by the laser vibrometer and sent back to material analyzer for processing. The tested samples had the same dimensions (2.5 × 2 × 0.25 mm). The S-E loops of the 3D printed sample and commercial sample are plotted and compared in Fig. S6b, showing that the 3D printed sample can generate near-pristine strain under the same electric field.

SI. 5 3D printable packaging material

The matching material was formed by mixing carbon fiber (CF) with RIGID resin (Formlab, USA). RIGID resin is the mixture of urethane dimethacrylate and isobornyl methacrylate. The acoustic

impedance of the matching material reached the values from 3 Mrayl to 6.7 Mrayl by tuning the matching layer's CF loading from 25 wt% to 45 wt%. The CF could increase the composite density and sound speed with a little increment of attenuation coefficient[6]. The thickness of the fabricated matching layer was designed as the one-quarter of the wavelengths of the transmitted ultrasound to reduce the acoustic loss[7].

The backing material is the Fe magnetic particle-loaded Flexible resin (Formlab, USA). Flexible is the mixture of acrylated oligomers and acrylated monomers. By tuning the Fe magnetic particle loading in the backing layer from 3 wt% to 20 wt%, the attenuation coefficient of the backing materials can range from -4.3 dB/mm to -10 dB/mm for 10-MHz sound waves damping. The Fe magnetic particle loading can increase sound dissipation by increase the thermal loss caused by the friction of particles[8]. The acoustic impedance of the backing material ranged from 2.6 Mrayl to 8 Mrayl, making it suitable for many transducer applications mentioned in the main text.

The design, material selection, and assembly of the matching layers depend on the intended applications and the function of the transducers. Using the transducers with 3-3 working modes as an example, which is also a commonly used ultrasonic transducer type for imaging and nondestructive testing, the matching layer is designed based on the shape of the aperture of the transducer. The material selection is determined by the sound propagation medium, as we mentioned in the manuscript. The thickness of matching layer was controlled to be less than one-fourth of the ultrasound wavelength to minimize the acoustic attenuation[9]. For our focused miniatured transducer, we 3D printed the matching layer to fit the size of the sintered piezo element and glue the matching layer with the piezo part using the same photocurable material used for the

matching layer. For the transducer with microarchitectures, such as the lattice transducers, we designed the matching layer as flat membranes and glue it onto the surface that receives sound wave.

SI. 6 Benchmark the energy output capability and sound beam lateral resolution of our 3D-printed transducer with the state-of-the-art

We benchmark our 3D-printed transducer with reported micro transducers in Table S1. Normalized p_{rms} is defined as the negative acoustic pressure generated by the element per unit voltage input per surface area and used to describe the energy output capability. For the studies that use acoustic intensity to characterize the performance of the transducers, we convert the spatial-peak-pulse-average intensity I_{sppa} into the root-mean-square acoustic pressure p_{rms} via[10]

$$p_{rms} = \sqrt{I_{sppa}\rho_o c} \qquad S6.1$$

where ρ_o is the density of sound propagation medium and c is the sound velocity in the medium. As shown in the table below, our transducer has the highest values (3.48 MPa (p_{rms} = 2.46 MPa) at 150 V_{pp}) for the normalized p_{rms} compared with other miniatured transducers.

Table S1 *Transducer energy output capability comparison*

Element geometry	Input signal	Working frequency (MHz)	Surface area (mm^2)	Normalized p_{rms} (kPa/V× mm^2)
Stacked plates[11]	Sin wave, 10% duty cycle	0.62	1.5×1.5	3.11
Plate[12]	Sin wave, 4% duty cycle	8.5	2 ×1.5	0.73
Stacked plates[13]	Sin wave, 10% duty cycle	1.92	2×2	2.83

Disk[14]	Sin wave, 10% duty cycle	5.3	16×π	0.28
Curved plate[15]	Sine wave, 2% duty cycle	11	2.5×7.5	0.18
Curved plate (This work)	Sin wave, 10% duty cycle	9.75	2.5×2	3.28

The lateral resolution of ultrasonic transducers is determined by the geometries and driving frequencies of piezo elements[16]. We simulated the acoustic intensity field of our transducer with COMSOL by inputting transducer aperture geometry and working frequency (Fig. S7a) and verified the result with experiments (Fig. S7c). The hydrophone (HGL-0200, ONDA, USA) was controlled by motors to capture the sound signal of the transducer, as shown in Fig. S7b. The resolution is defined by the -6 dB threshold of the acoustic intensity field. The simulated and measured resolutions of our transducer are 0.08 mm and 0.18 mm, respectively. Compared with the simulated results of the reported transducers (Fig. S7d, S7e and S7f), our transducer has the highest lateral resolution owing to its focusing feature and high working frequency.

SI. 7 Energy output performance variations of the ultrasound transducers along with the shapes

To demonstrate the performance of the elements with complex geometries, we fabricated disk elements (Fig. S8b) and elements with concentric annular layers (Fig. S8c) and packaged them with the same procedure and material as the curved elements (matching layer (CF (28wt%) - matching layer composite) and backing layer (Fe (3wt%) - backing layer composite). The element with concentric annular layers was filled with epoxy, which can suppress the transverse vibration of the elements[17]. The acoustic pressure was measured using standard hydrophone (HGL-0400, ONDA, USA) by placing it at the focal area[18] of the transducers, as shown in Fig. S8a. The negative

acoustic pressure of the as-fabricated transducers is plotted in Fig. S8d. The disk element can generate the negative acoustic pressure of 0.86 MPa under the voltage of 150 V_{pp}, while the negative acoustic pressure produced by element with concentric layers can be up to 0.99 MPa. Finite element analysis (COMSOL, USA) was used to show the generated acoustic field of the transducers (Fig. S8e and S8f).

SI. 8 Dye diffusion coefficient and microbubble concentration

Our transducer achieves cavitation assisted diffusion of molecules. Here, we tested the efficiency in the diffusion of methylene blue, a salt used as a dye and as a medication in aqueous solutions. We prepared the experiment by gently dropping methylene blue into aqueous solution and measuring its diffusion coefficient[19] with and without the assistance of microbubble cavitation at 90 V_{pp} (Supplementary Movie 2). The container was divided into six sections. The change of their pixel value standard deviation was used to evaluate the diffusion uniformity (Fig. S11). The ultrasound radiation force-assisted diffusion and cavitation-assisted diffusion can be calculated based on the methylene blue diffusion coefficient in the water[19].

The methylene blue in both the water and microbubble suspensions started to disperse, as shown in Fig. S12 and Supplementary Movie 2. The dispersion in the microbubble suspension rapidly began when the cavitation threshold was reached, and the corresponding standard deviations decreased faster than that in the tap water group. At the 15th second of the ultrasound exposure, methylene blue in the microbubble suspension was fully dissolved in the solution, and the standard deviation was decreased by 97% compared to its original value, while the methylene blue in tap

water was mainly dispersed in the bottom part with a 21% drop in the standard deviation. With the assistance of cavitation, the diffusion coefficient reached 36.2 × 10^{-5} cm^2/s, which is 6.4 times of the value calculated for the tap water group. Without cavitation, both the diffusion coefficient and pixel standard deviation change in the microbubble suspension remained the same as those in the tap water group, showing that the microbubble suspension alone has no effect on the dye dispersion.

In localized cavitation experiment, microbubble solution with different concentrations was injected into the transparent blood vessel phantom. The pixel value - microbubble concentration digital imaging correlation (DIC) curve was plotted in Fig. S13. The microbubble fragmentation speeds were achieved by correlating frames of experiment videos at different times and the DIC curve.

References

1. Walker, D. A., Hedrick, J. L. & Mirkin, C. A. Rapid, large-volume, thermally controlled 3D printing using a mobile liquid interface. *Science* **366**, 360–364 (2019).
2. Cui, H. *et al.* Three-dimensional printing of piezoelectric materials with designed anisotropy and directional response. *Nature Mater* **18**, 234–241 (2019).
3. Yao, D. *et al.* Achieving the Upper Bound of Piezoelectric Response in Tunable, Wearable 3D Printed Nanocomposites. *Advanced Functional Materials* **29**, 1903866 (2019).
4. Dukhin, A. S. & Goetz, P. J. Chapter 7 - Acoustic and Electroacoustic Measurement Techniques. in *Studies in Interface Science* (eds. Dukhin, A. S. & Goetz, P. J.) vol. **24** 261–301 (Elsevier, 2010).

5. Dukhin, A. S. & Goetz, P. J. Chapter 3 - Fundamentals of Acoustics in Homogeneous Liquids: Longitudinal Rheology. in *Studies in Interface Science* (eds. Dukhin, A. S. & Goetz, P. J.) vol. **24** 91–125 (Elsevier, 2010).
6. Yan, Z., Pu, Z., Haijun, F. & Yi, Z. Experiment Study on Sound Properties of Carbon Fiber Composite Material. *IOP Conference Series: Materials Science and Engineering* **542**, 012001 (2019).
7. Shung, K. K. & Zippuro, M. Ultrasonic transducers and arrays. *IEEE Engineering in Medicine and Biology Magazine* **15**, 20–30 (1996).
8. Cao, L., Fu, Q., Si, Y., Ding, B. & Yu, J. Porous materials for sound absorption. *Composites Communications* **10**, 25–35 (2018).
9. Zhou, Q., Lam, K. H., Zheng, H., Qiu, W. & Shung, K. K. Piezoelectric single crystals for ultrasonic transducers in biomedical applications. *Prog Mater Sci* **66**, 87–111 (2014).
10. Mickiewicz, W., Raczyński, M. & Parus, A. Performance Analysis of Cost-Effective Miniature Microphone Sound Intensity 2D Probe. *Sensors (Basel, Switzerland)* **20**, (2020).
11. Kim, J. *et al.* Intravascular forward-looking ultrasound transducers for microbubble-mediated sonothrombolysis. *Sci Rep* **7**, 3454 (2017).
12. Shih, C.-C. *et al.* Development of an intravascular ultrasound elastography based on a dual-element transducer. *R. Soc. open sci.* **5**, 180138 (2018).
13. Kim, H., Wu, H., Goel, L. & Jiang, X. Miniaturized Ultrasound Transducer Composed of a Composite of Multiple Piezoelectric Stacks. in (American Society of Mechanical Engineers Digital Collection, 2020). doi:10.1115/IMECE2019-12208.
14. Lim, H. G. *et al.* Thermal Ablation and High-Resolution Imaging Using a Back-to-Back (BTB) Dual-Mode Ultrasonic Transducer: In Vivo Results. *Sensors* **21**, 1580 (2021).

15. Bouchoux, G. *et al.* Dual-Mode Ultrasound Transducer for Image-Guided Interstitial Thermal Therapy. *Ultrasound in Medicine & Biology* **34**, 607–616 (2008).

16. Basak, A. & Bhunia, S. Chapter 13 - Implantable imaging system for automated monitoring of internal organs. in *Implantable Biomedical Microsystems* (eds. Bhunia, S., Majerus, S. J. A. & Sawan, M.) 281–312 (William Andrew Publishing, 2015). doi:10.1016/B978-0-323-26208-8.00013-3.

17. Hu, H. *et al.* Stretchable ultrasonic transducer arrays for three-dimensional imaging on complex surfaces. *Science Advances* **4**, eaar3979 (2018).

18. Sadeghi Gougheri, H., Dangi, A., Kothapalli, S.-R. & Kiani, M. A Comprehensive Study of Ultrasound Transducer Characteristics in Microscopic Ultrasound Neuromodulation. *IEEE Trans Biomed Circuits Syst* **13**, 835–847 (2019).

19. Selifonov, A. A., Shapoval, O. G., Mikerov, A. N. & Tuchin, V. V. Determination of the Diffusion Coefficient of Methylene Blue Solutions in Dentin of a Human Tooth using Reflectance Spectroscopy and Their Antibacterial Activity during Laser Exposure. *Optics and Spectroscopy* **126**, 758–768 (2019).

20. Chaussy, C., Tilki, D. & Thüroff, S. Transrectal High-Intensity Focused Ultrasound for the Treatment of Localized Prostate Cancer: Current Role. **2013**, (2013).

Supplementary Figures

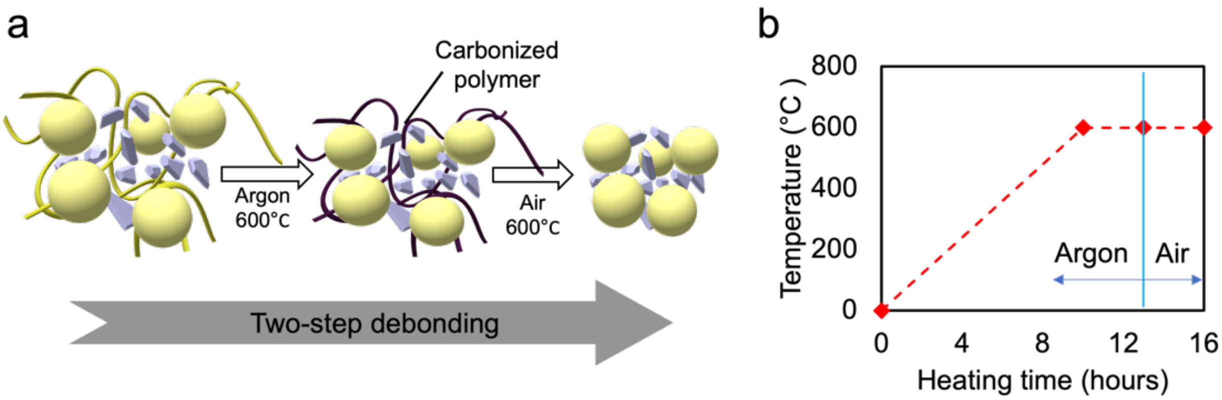

Figure S1 Debonding of the 3D-printed PZT. a) Two-step debonding process. **b**) Debonding time-temperature curve.

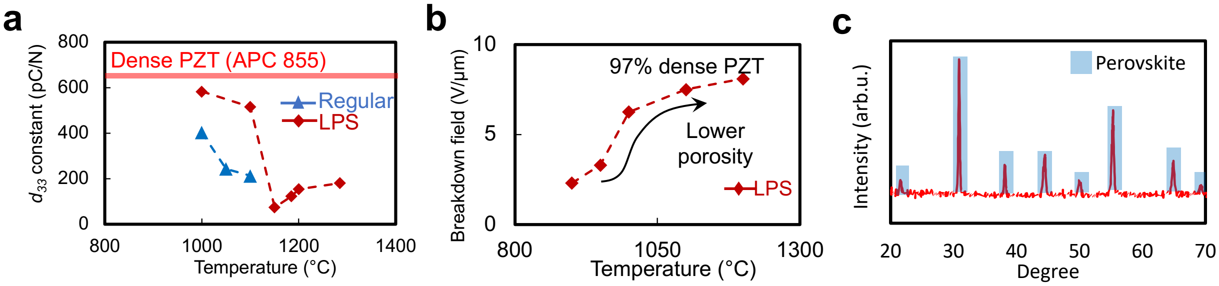

Figure S2 Sintering temperature adjusting of 3D printed PZT. Peak sintering temperature optimized by **a**) d_{33} constant and **b**) Breakdown electrical field of the samples. **c**) XRD image of the sintered PZT sample.

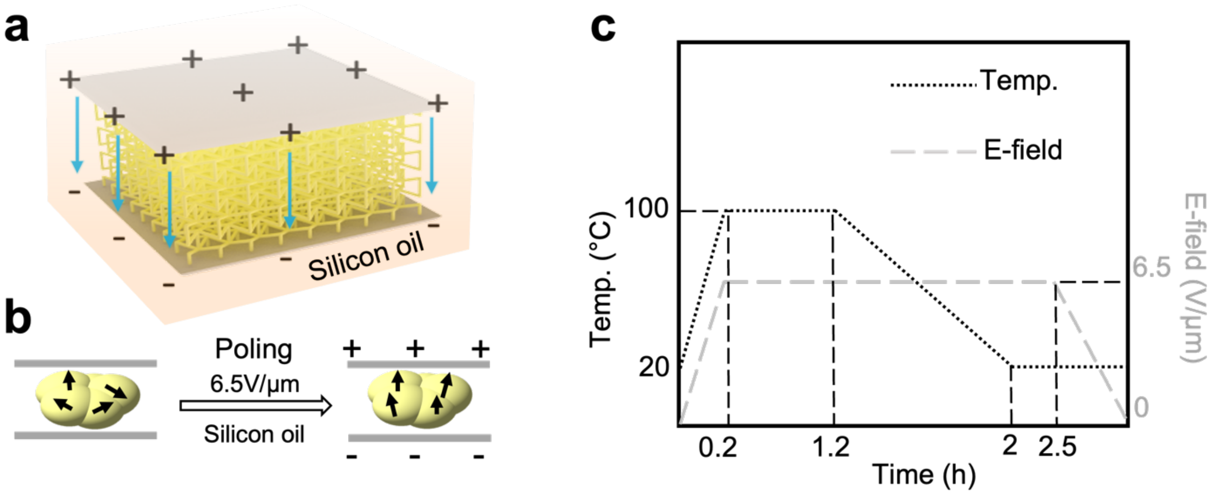

Figure S3 Polarization of the 3D printed PZT. a) Schematic of polarization setup. **b**) Schematic of the dipole alignment along the applied electrical field. **c**) Polarization time and electrical field profile.

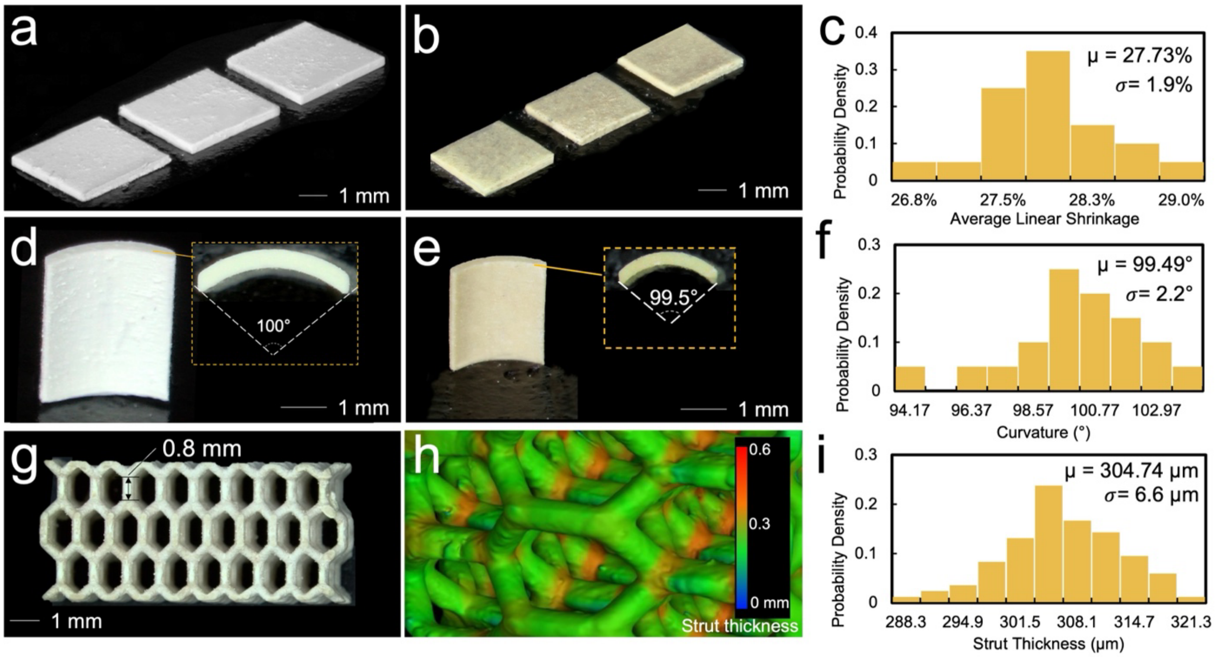

Figure S4 Structural integrity measurement of sintered samples. **a**) and **b**) 3D printed rectangular samples before and after sintering, showing their dimension shrinkages were identical after sintering process. **d**) and **e**) 3D printed sample with focusing feature, showing the curvature were well maintained during sintering. **g**) 3D printed honeycomb lattice. **h**) XCT image of the unit cell of the lattice sample. **c**), **f**) and **i**) are probability distribution of average linear shrinkage ratio, curvature and struct thickness. μ is the mean value while σ is the standard deviation.

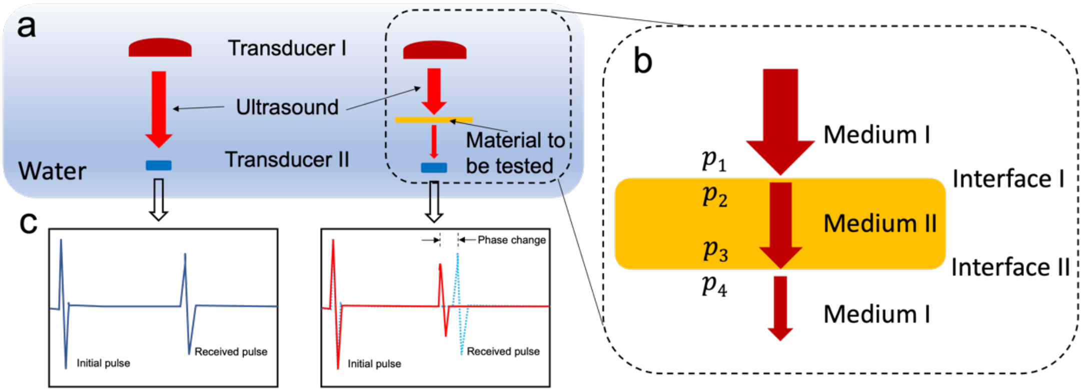

Figure S5 Sound speed within the material measurement. a) Experiment set up for sound speed and acoustic attenuation measurement. **b**) Acoustic pressures near and within the sample to be tested (*p*: acoustic pressure) **c**) Schematics of the received signals with and without sample in between transducers.

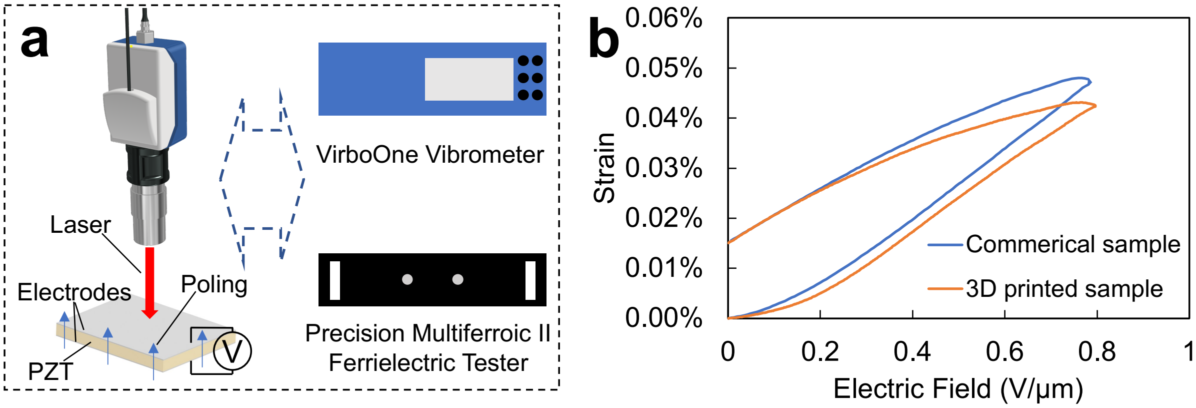

Figure S6 Strain-Electric field loop measurement of commercial sample and 3D printed sample. **a**) Schematic of the S-E loop testing system. **b**) S-E curves of the commercial PZT sample and 3D printed sample.

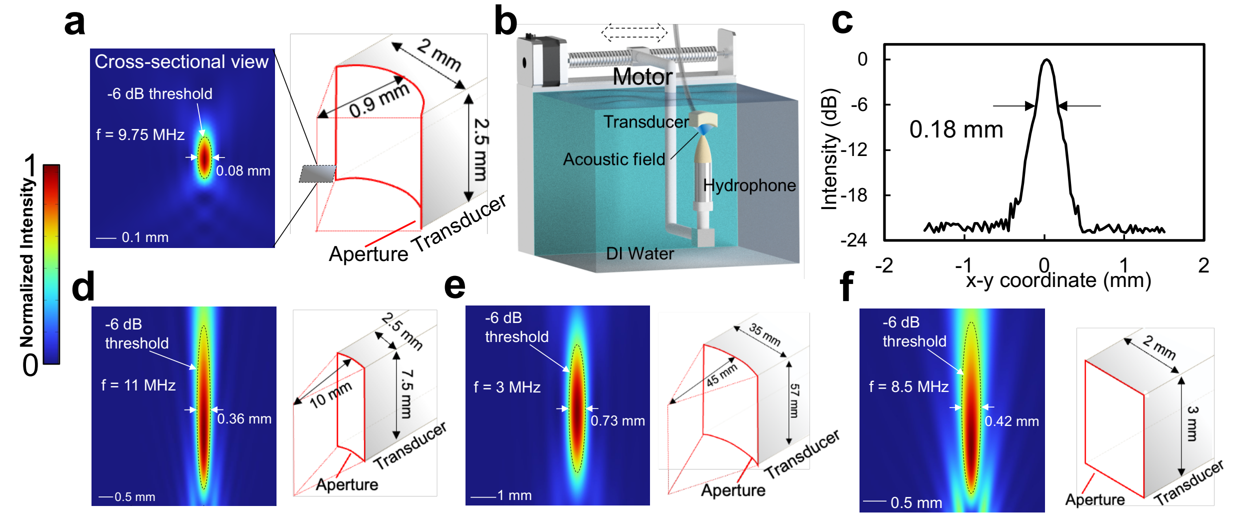

Figure S7 Micro transducer acoustic field simulation and experimental verification. a) Acoustic field simulation of the as-fabricated transducer. **b**) Schematic of the experimental setup for measuring transducer resolution. **c**) Experimental results of the signal magnitude of the as-fabricated transducer. **d-f**) Acoustic field simulation of the reported transducers[12,15,20]

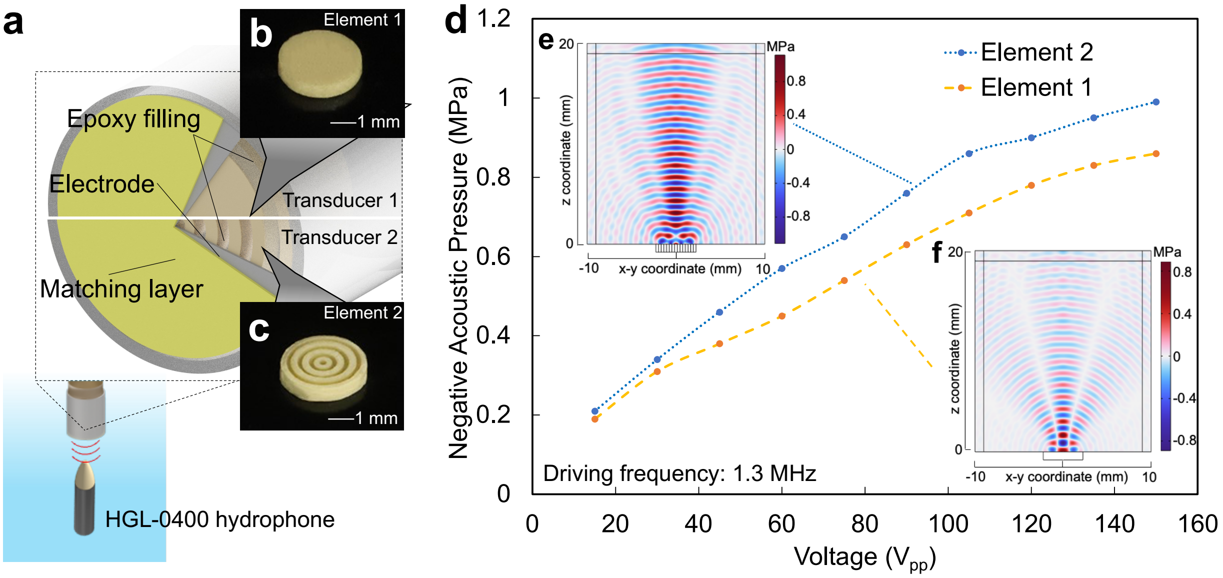

Figure. S8 3D printed transducers of different geometries and their energy output capability. **a**) Schematic of the transducer packaging, and the schematic of the acoustic pressure measurement setup. **b-c**) Optical images of the disk element and the element with concentric annular layers. **d**) Peak-to-peak acoustic pressure generated by the transducers. **e-f**) FEA models to predict the acoustic pressure and the acoustic fields generated by the transducers.

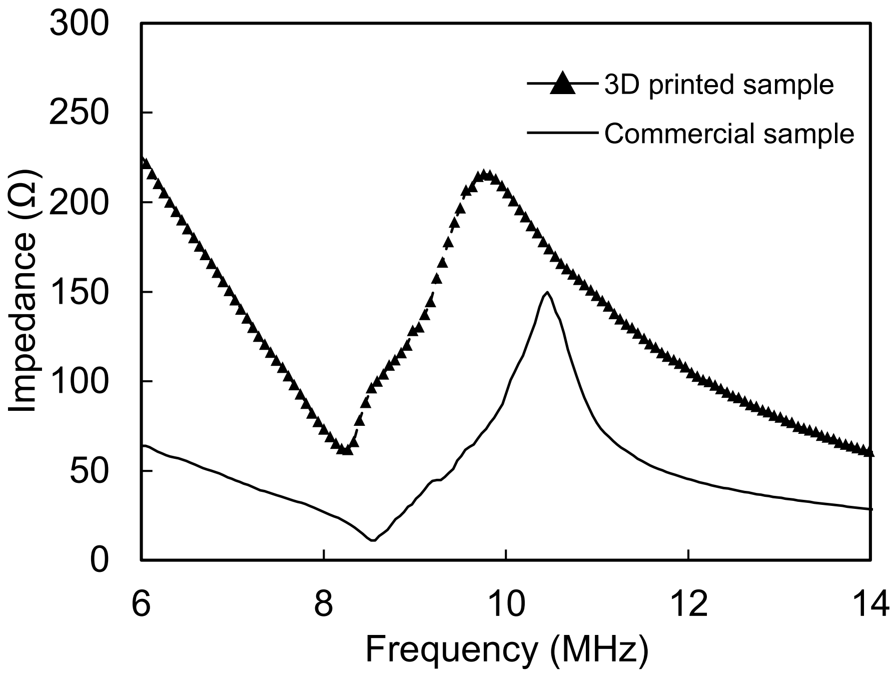

Figure S9 Impedance curves of the fabricated PZT sample and standard PZT sample.

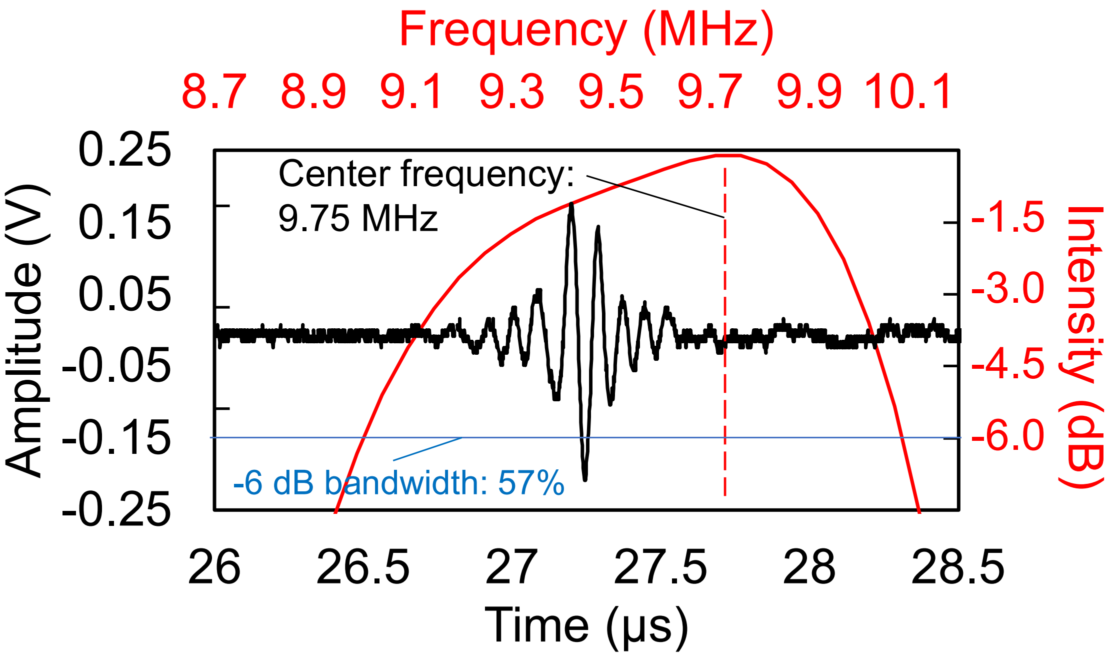

Figure S10. **3D printed miniatured ultrasound transducer pulse-echo signal and its frequency spectrum.**

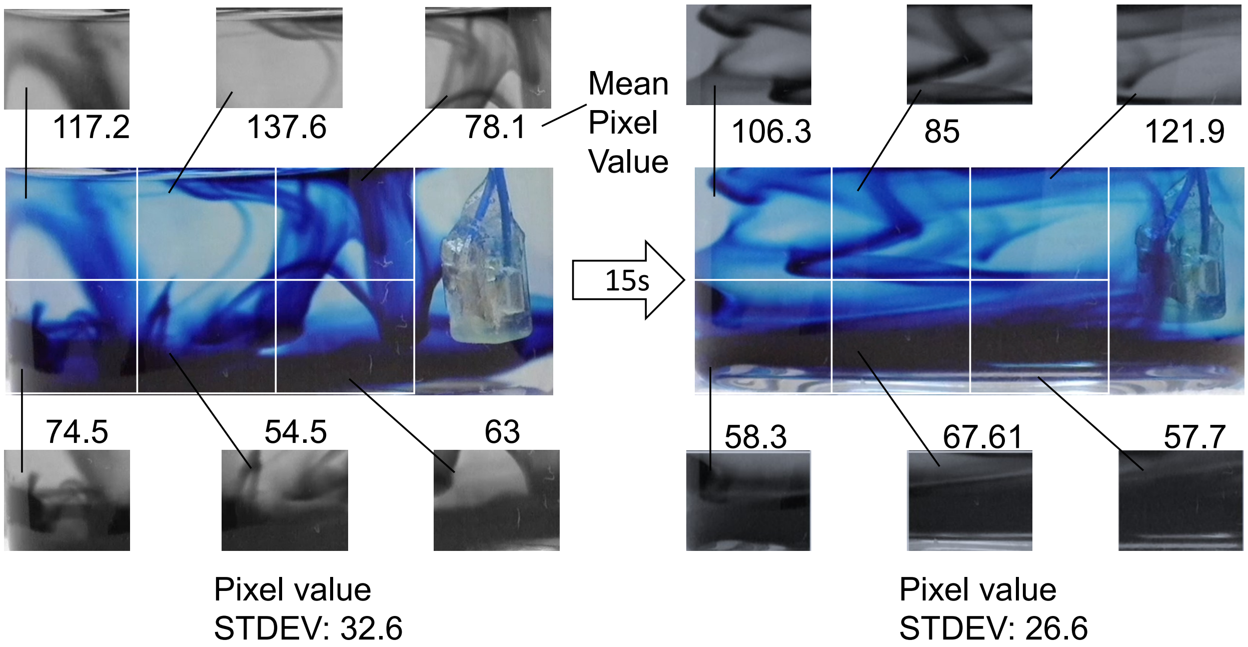

Figure S11. Dye diffusion speed and uniformity calculation based on the pixel value of grayscale images.

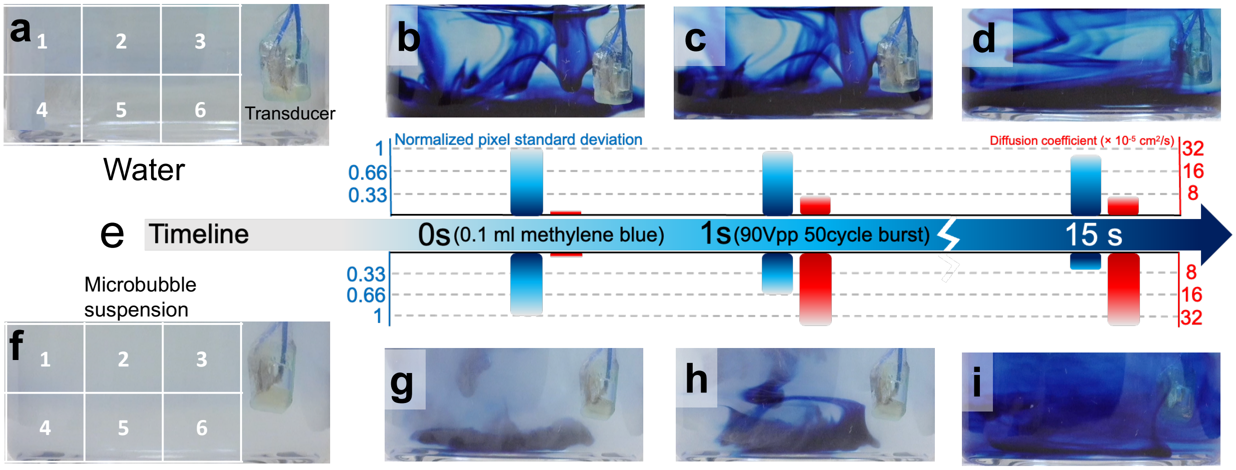

Figure S12 Dye diffusion speed and uniformity calculation based on the pixel value of grayscale images. a), **f**) Dye dispersion experiment set up in water and in a microbubble suspension. **b**), **g**) A 0.5 ml methylene blue drop was added to both media. **c**), **h**) 9.75-MHz burst waves were input into the transducer, and the dye was stirred by the emitted acoustic wave. **d**), **i**) After 15 seconds of ultrasound exposure, the dye partially dispersed in the water but fully dispersed in the microbubble suspension. **e**) Timeline of the changes in the normalized pixel value standard deviations and diffusion coefficients of both water and microbubble groups.

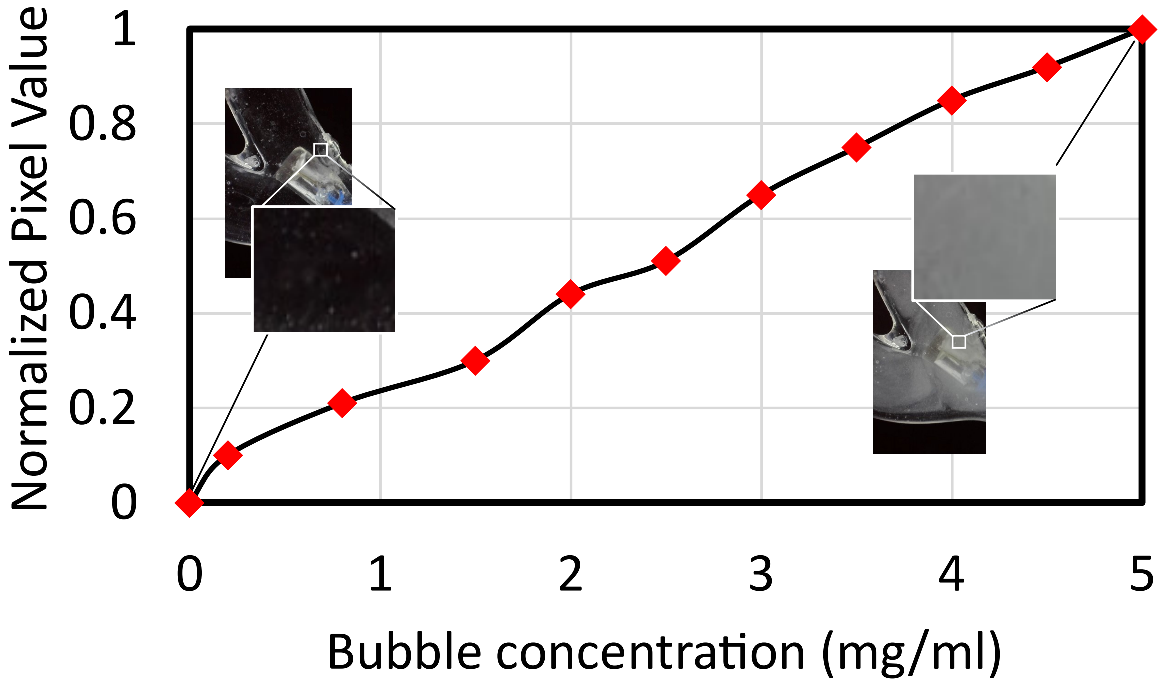

441 **Figure S13 Digital image correlation curve of microbubble concentration in the blood vessel**

442 **phantom vs. image mean pixel value.**
