# Supplementary material for: 3D Printing and processing of miniaturized transducers with near-pristine piezoelectric ceramics for localized cavitation
Source: Nat Commun. 2023 Apr 27;14:2418. doi: 10.1038/s41467-023-37335-w (PMC10140030; doi:10.1038/s41467-023-37335-w)
Supplement: Supplementary file 2 — Description of Additional Supplementary Files [file 41467_2023_37335_MOESM2_ESM.pdf]

Description of Additional Supplementary Files

File Name: Supplementary Movie 1
Description: Microbubble behaviour during insonation

File Name: Supplementary Movie 2
Description: Cavitation-assisted dye diffusion.

File Name: Supplementary Movie 3
Description: Localized cavitation in blood vessel phantom.

File Name: Supplementary Movie 4
Description: Localized cavitation on the wall of the blood vessel phantom.
