# Supplementary material for: 3D Printing and processing of miniaturized transducers with near-pristine piezoelectric ceramics for localized cavitation
Source: Nat Commun. 2023 Apr 27;14:2418. doi: 10.1038/s41467-023-37335-w (PMC10140030; doi:10.1038/s41467-023-37335-w)
Supplement: Supplementary file 7 — Reporting Summary [file 41467_2023_37335_MOESM7_ESM.pdf]

nature portfolio

Corresponding author(s): Xiaoyu (Rayne) Zheng

Last updated by author(s): Mar 1, 2023

Reporting Summary

Nature Portfolio wishes to improve the reproducibility of the work that we publish. This form provides structure for consistency and transparency in reporting. For further information on Nature Portfolio policies, see our Editorial Policies and the Editorial Policy Checklist.

Statistics

For all statistical analyses, confirm that the following items are present in the figure legend, table legend, main text, or Methods section.

n/a	Confirmed	
☐	☒	The exact sample size (*n*) for each experimental group/condition, given as a discrete number and unit of measurement
☐	☒	A statement on whether measurements were taken from distinct samples or whether the same sample was measured repeatedly
☒	☐	The statistical test(s) used AND whether they are one- or two-sided<br>*Only common tests should be described solely by name; describe more complex techniques in the Methods section.*
☒	☐	A description of all covariates tested
☒	☐	A description of any assumptions or corrections, such as tests of normality and adjustment for multiple comparisons
☐	☒	A full description of the statistical parameters including central tendency (e.g. means) or other basic estimates (e.g. regression coefficient) AND variation (e.g. standard deviation) or associated estimates of uncertainty (e.g. confidence intervals)
☒	☐	For null hypothesis testing, the test statistic (e.g. *F*, *t*, *r*) with confidence intervals, effect sizes, degrees of freedom and *P* value noted<br>*Give P values as exact values whenever suitable.*
☒	☐	For Bayesian analysis, information on the choice of priors and Markov chain Monte Carlo settings
☒	☐	For hierarchical and complex designs, identification of the appropriate level for tests and full reporting of outcomes
☒	☐	Estimates of effect sizes (e.g. Cohen's *d*, Pearson's *r*), indicating how they were calculated

Our web collection on statistics for biologists contains articles on many of the points above.

Software and code

Policy information about availability of computer code

Data collection	We collected the experimental data using Vision 3 and Vision 5, which are available in Radiant Technologies, Inc.
Data analysis	We analyzed the data using MATLAB 2021b and COMSOL Multiphysics 6.0.

For manuscripts utilizing custom algorithms or software that are central to the research but not yet described in published literature, software must be made available to editors and reviewers. We strongly encourage code deposition in a community repository (e.g. GitHub). See the Nature Portfolio guidelines for submitting code & software for further information.

Data

Policy information about availability of data

All manuscripts must include a data availability statement. This statement should provide the following information, where applicable:

- Accession codes, unique identifiers, or web links for publicly available datasets
- A description of any restrictions on data availability
- For clinical datasets or third party data, please ensure that the statement adheres to our policy

All data are included in the manuscript and the supplementary information.

Human research participants

Policy information about studies involving human research participants and Sex and Gender in Research.

Reporting on sex and gender	No human research participants were involved in this study.
Population characteristics	See above
Recruitment	No human research participants were involved in this study.
Ethics oversight	No human research participants were involved in this study.

Note that full information on the approval of the study protocol must also be provided in the manuscript.

Field-specific reporting

Please select the one below that is the best fit for your research. If you are not sure, read the appropriate sections before making your selection.

☐ Life sciences ☐ Behavioural & social sciences ☐ Ecological, evolutionary & environmental sciences

For a reference copy of the document with all sections, see nature.com/documents/nr-reporting-summary-flat.pdf

Life sciences study design

All studies must disclose on these points even when the disclosure is negative.

Sample size	In this study, no life sciences researches were involved.
Data exclusions	In this study, no life sciences researches were involved.
Replication	In this study, no life sciences researches were involved
Randomization	In this study, no life sciences researches were involved.
Blinding	In this study, no life sciences researches were involved.

Behavioural & social sciences study design

All studies must disclose on these points even when the disclosure is negative.

Study description	In this study, no behavioural & social sciences researches were involved.
Research sample	In this study, no behavioural & social sciences researches were involved.
Sampling strategy	In this study, no behavioural & social sciences researches were involved.
Data collection	In this study, no behavioural & social sciences researches were involved.
Timing	In this study, no behavioural & social sciences researches were involved.
Data exclusions	In this study, no behavioural & social sciences researches were involved.
Non-participation	In this study, no behavioural & social sciences researches were involved.
Randomization	In this study, no behavioural & social sciences researches were involved.

Ecological, evolutionary & environmental sciences study design

All studies must disclose on these points even when the disclosure is negative.

Study description	No ecological, evolutionary & environmental sciences studies were involved
Research sample	No ecological, evolutionary & environmental sciences studies were involved
Sampling strategy	No ecological, evolutionary & environmental sciences studies were involved
Data collection	No ecological, evolutionary & environmental sciences studies were involved
Timing and spatial scale	No ecological, evolutionary & environmental sciences studies were involved
Data exclusions	No ecological, evolutionary & environmental sciences studies were involved
Reproducibility	No ecological, evolutionary & environmental sciences studies were involved
Randomization	No ecological, evolutionary & environmental sciences studies were involved
Blinding	No ecological, evolutionary & environmental sciences studies were involved

Did the study involve field work? ☐ Yes ☒ No

Reporting for specific materials, systems and methods

We require information from authors about some types of materials, experimental systems and methods used in many studies. Here, indicate whether each material, system or method listed is relevant to your study. If you are not sure if a list item applies to your research, read the appropriate section before selecting a response.

Materials & experimental systems

n/a	Involved in the study	
☒	☐	Antibodies
☒	☐	Eukaryotic cell lines
☒	☐	Palaeontology and archaeology
☒	☐	Animals and other organisms
☒	☐	Clinical data
☒	☐	Dual use research of concern

Methods

n/a	Involved in the study	
☒	☐	ChIP-seq
☒	☐	Flow cytometry
☒	☐	MRI-based neuroimaging
